# Effectiveness of a family violence prevention program on mental health outcomes for adult men and women in North Kivu, Democratic Republic of Congo: Insights from a pilot trial

**DOI:** 10.1017/gmh.2024.98

**Published:** 2024-10-24

**Authors:** Manya Balachander, Jean de Dieu Hategekimana Ndiyunze, Danielle Roth, Khudejha Asghar, Christine Bourey, Kathryn L. Falb

**Affiliations:** 1 Department of International Health, Johns Hopkins Bloomberg School of Public Health, Baltimore, Maryland, USA; 2 Women’s Protection and Empowerment, International Rescue Committee, Goma, North Kivu, Democratic Republic of Congo; 3 Violence Prevention and Response, International Rescue Committee, Washington, DC, USA; 4 University of Edinburgh School of Social and Political Science, Edinburgh, USA; 5 Columbia University Mailman School of Public Health, New York, NY, USA

**Keywords:** depression, intimate partner violence, child maltreatment, humanitarian, randomized controlled trial

## Abstract

The eastern Democratic Republic of Congo (DRC) has faced dual burdens of poor mental health and heightened levels of violence against women and children within the home. Interventions addressing family violence prevention may offer a path to mitigate mental distress within the eastern DRC. This exploratory analysis uses data from a pilot cluster randomized controlled trial conducted in North Kivu, DRC, assessing the impact of Safe at Home, a violence prevention intervention. Mental health was assessed at endline using the Patient Health Questionnaire-4. Statistical analyses employed multilevel linear regression.

Assuming successful randomization, impact of the Safe at Home intervention on mental health differed by participant gender. Women enrolled in the Safe at Home intervention reported statistically significant decreases in mental distress symptoms [β (95%CI) = −1.01 (−1.85, −0.17)], whereas men enrolled in Safe at Home had similar scores in mental distress to the control group [β (95%CI) = −0.12 (−1.32, 1.06)].

Ultimately, this exploratory analysis provides evidence of the potential for a family violence prevention model to improve women’s mental health in a low-resource, conflict-affected setting, although further research is needed to understand the impact on men’s mental health.

## Impact statement

The eastern Democratic Republic of Congo (DRC) has endured prolonged armed conflict, significantly affecting mental health. Conflict-related stressors have also been reported to exacerbate family violence, compounding mental health challenges. Preventing family violence could serve as a potential avenue to alleviate mental distress within families. This study uses data from a previously completed pilot cluster randomized controlled trial to assess the potential impact of the Safe at Home family strengthening and feminist-grounded violence prevention intervention on the mental health of adult participants in four conflict-affected villages in North Kivu, DRC. The results indicated a statistically significant improvement in mental health for women enrolled in the Safe at Home intervention, while men’s mental health enrolled in the intervention showed a positive trend without reaching statistical significance. This suggests that conflict-affected women’s mental health may be improved when family relationships are strengthened, but these findings did not fully translate for men. These insights should be interpreted with limitations in mind, including a small sample size, a lean, generalized measure of mental health and lack of baseline mental health assessment. Nonetheless, this analysis points to the potential importance of violence prevention strategies that grapple with power inequities within the home in humanitarian settings as an avenue for future longitudinal and intervention research to improve mental health.

## Introduction

Globally, an estimated one in eight people have a mental illness (WHO, 2022). Within humanitarian settings, the prevalence of mental distress, particularly anxiety and major depressive disorders (MDDs), is estimated to be two to four times higher than global prevalence estimates (Charlson et al., 2019). High prevalence of post-traumatic stress disorder (PTSD) within conflict settings is also well documented in the literature (de Jong et al., 2001; Charlson et al., 2019; Seidi and Jaff, 2019), and mental health concerns commonly co-occur (Hoppen et al., 2021). A multitude of factors across the ecological model may exacerbate the risk of poor mental health. These may include individual direct experiences of conflict-related traumas and loss of livelihoods, alongside family-level factors such as displacement and separation from family, among other factors, including a breakdown of protective institutions and norms (Miller and Rasmussen, 2010). Within the eastern Democratic Republic of Congo (DRC), which has faced vacillating levels of armed conflict and other crises for over 20 years, studies have documented mental health concerns. For example, previous research has shown that two of five adults meet the criteria for MDD and half for PTSD (Johnson et al., 2010). These heightened mental health concerns have been found to be associated with human rights abuses and conflict-related sexual violence against women (Johnson et al., 2010; Dossa et al., 2015). Combined, the evidence demonstrates an explicit need for mental health care among conflict-afflicted areas of the DRC (On’okoko et al., 2010).

Beyond experiences related to conflict and widespread exposure to traumas that may produce poor mental health, exposure to violence within the home may also result in suboptimal mental health for women. These factors may interact with other drivers of violence against women in humanitarian settings, such as a breakdown in protective institutions, changing gender norms and challenges in meeting basic needs to perpetuate and reproduce violence against women (Wachter et al., 2018; Murphy et al., 2023). Findings mirror other conflict-affected settings that have documented poor mental health and intimate partner violence (Devries et al., 2013; Ellsberg and Emmelin, 2014; Silove et al., 2017). The relationship between violence exposure and adverse mental health outcomes can operate in several directions: violence exposure may exacerbate mental distress (Lagdon et al., 2014), mental distress may increase violence exposure (Trevillion et al., 2012); and cyclically, violence exposure may exacerbate mental distress, which may increase violence exposure and, in turn, worsen mental distress (Spencer et al., 2019).

Indeed, the relationship between violence, traumas and poor mental health are interconnected and complex in conflict-affected settings. For example, previous qualitative research in eastern DRC documented men’s experience of trauma related to armed conflict may interact with inequitable gender norms and other drivers to exacerbate their use of violence in the home (Falb et al., 2022). Similar findings have been documented in other populations, such as among United States military veterans (Taft et al., 2005), in Timor-Leste (Rees et al., 2018), in Liberia (Vinck and Pham, 2013), in South Africa (Gupta et al., 2012), and qualitatively in Cote d’Ivoire (Falb et al., 2014). Furthermore, even residing in areas highly affected by armed conflict may also lead to long-term risk of IPV perpetration by men (Gibbs et al., 2020). Analyses guided in hegemonic masculinity theory suggest that trauma exposure may be linked to increased blame externalization among men, manifesting in more controlling behavior and engagement in violent behaviors, including rape (Jewkes and Morrell, 2018). This behavior may be particularly enhanced in conflict-affected settings, where the unique stressors of conflict settings manifest in post-traumatic stress symptoms.

For women, experiencing trauma and reporting suboptimal mental health has also been linked to increased risk of experiencing violence in the home in conflict-affected settings (Kelly et al., 2011; Rees et al., 2011; Devries et al., 2013). The majority of this literature suggests norms around family honor and shame may further marginalize women who have experienced violence during conflict, and in particular, sexual violence (Dietrich and Schuett, 2013; Brown et al., 2018). This social exclusion results in limited access to social support (Meinhart et al., 2022) and potential exclusion from economic opportunities, which limit a woman’s marriage pool and ability to leave an abusive partner (García-Moreno, 2014; Singh et al., 2022). Additionally, the experience of recent IPV, even within conflict-affected settings, has also been associated with increased levels of poor mental health, even beyond traumatic experiences related to conflict (Falb et al., 2013, 2019).

From an intervention standpoint, given the complex and interconnected relationship between violence, trauma and poor mental health, it is plausible for family violence prevention programs within humanitarian settings to impact the mental health of its participants. Common elements of standard violence prevention programming may include communication and decision-making strategies, power sharing and parenting skills and efforts to address inequitable attitudes, alongside increased referrals and safety planning opportunities for women experiencing violence. Within these programs, women may have improvements in mental health through these mechanisms, alongside potential reductions in violence victimization. For men, mental health may also improve because interventions build resilience by strengthening positive masculine models, improving emotion regulation and building stress management skills. Indeed, studies exploring the role of violence prevention programs on mental health outcomes have found that implementing violence prevention and educational programs in LMICs can improve mental health outcomes for those populations impacted by violence (Butchart et al., 2015; Cluver et al., 2016, 2020). Research exploring the role of mental health and psychosocial support interventions in humanitarian settings has repeatedly underscored the importance of violence prevention and safety programs, such as Child Friendly Spaces, community-initiated social support and group-based support (Tol et al., 2011; Stark et al., 2021). This further emphasizes the potential importance of violence prevention programs as a mechanism through which mental distress can be minimized, and this association should be further explored.

One such intervention, Safe at Home, is a violence prevention program developed to improve family well-being and prevent multiple forms of violence in the home. Safe at Home was piloted in North Kivu to understand its impact on family functioning, co-occurring violence, IPV and harsh discipline (Falb et al., 2023a). The pilot trial found that Safe at Home was highly effective in preventing experiences of IPV and perpetrating harsh discipline among women as compared to the control; the pilot trial also found that Safe at Home was effective, in mitigating IPV perpetration among men as compared to the control (Falb et al., 2023b). The pilot additionally improved attitudes (e.g., non-acceptable of harsh discipline, gender-equitable attitudes) and skills (e.g., power sharing, positive parenting strategies) within couples, although findings were minimal for family functioning. Nonetheless, it represents an opportunity to explore the unintended consequences of how a family violence prevention program may also improve mental health and addresses an emerging field of research. Therefore, the objective of this exploratory analysis is to better understand the impact of Safe at Home, a family violence prevention intervention, implemented in North Kivu, DRC, on the mental health outcomes of the men and women in the couple who participated in the intervention.

## Methods

### Study design

This study employed a cluster randomized controlled trial in four villages in North Kivu, DRC: Kingarame, Kalangala, Buhumba and Munigi. North Kivu has experienced instability over the last few decades due to several contextual factors, including the historical legacies of Belgium colonial rule, spillover from the 1994 Rwandan genocide, and increasing numbers of conflict-displaced people and outbreaks across the region. From the study population, eight groups of participants were formed based on logistical factors, such as travel distance and time availability for participants and facilitators. Half of the groups (n = 4) were randomly allocated to receive the Safe at Home intervention, and half were allocated to a waitlisted comparison arm after endline data collection was completed (n = 4). Baseline data collection occurred between November 2019 and January 2020, and endline data collection occurred between August and September 2021. The full study details and the CONSORT diagram are provided in Falb et al., 2023b.

### Intervention

The Safe at Home (IRC, n.d.) program intervention has been extensively described elsewhere (Falb et al., 2023a) and includes weekly single-sex discussion groups with coupled men and women and monthly family discussion groups with children and couples. It was originally developed from in-depth qualitative research done in the DRC and Myanmar and was further piloted and refined in the DRC. Sessions occurred over the course of 6–8 months. During the single-sex sessions, men and women engaged separately on topics related to gender and age power hierarchies within the home and skill building related to communication, stress management and positive parenting. Based on in-depth design work with communities and lessons from a previous IPV prevention trial (Vaillant et al., 2020), single-sex sessions were selected to minimize men’s dominance of discussions in couple’s discussions. However, monthly couple and family sessions complemented these sessions and focused on improving relationship quality and decision-making, including children’s involvement for decisions, within the family. These integrated parenting approaches and trauma-informed stress management techniques had also been previously tested by International Rescue Committee (IRC) in Liberia and Thailand within their parenting programs (Sim et al., 2014; Puffer et al., 2015). The Safe at Home program was found to be highly efficacious in preventing men’s use of IPV against their partners (OR = 0.15; p = 0.000) and female’s use of harsh discipline against their children (OR = 0.29; p = 0.013) with downward trends of male’s use of harsh discipline (OR = 0.56; p = 0.19). Significant changes were also found for improvement in equitable attitudes and skills, but nonsignificant changes in family functioning (Falb et al., 2023b). The Safe at Home program was found to be highly efficacious in preventing men’s use of IPV against their partners (OR = 0.15; p = 0.000) and female’s use of harsh discipline against their children (OR = 0.29; p = 0.013) with downward trends of male’s use of harsh discipline (OR = 0.56; p = 0.19). Significant changes were also found for improvement in equitable attitudes and skills, but nonsignificant changes in family functioning (Falb et al., 2023b).

### Participants

In total, 202 couples (n = 404) were recruited from communities and screened for eligibility by the IRC. Eligibility criteria included: 18 years of age or older, living with a partner in a heterosexual, monogamous relationship who was also interested in participating, a speaker of Swahili, Kinyarwanda or French, and having at least one child between the ages of 6 and 12 years of age. At baseline, 394 (97.5%) men and women completed the survey, and at the endline, 391 (96.8%) completed the survey.

### Data collection and measures

The primary outcome of this analysis was the Patient Health Questionnaire-4 (PHQ-4) (Kroenke et al., 2009), which the investigators selected as a lean measure of mental health given it was an exploratory outcome of the program. It was only assessed at endline. The PHQ-4 is a well-validated measure of depression and anxiety symptoms (Löwe et al., 2010) that can screen for core symptoms of depression and anxiety. The questions ask: “Over the *last 2 weeks*, how often have you been bothered by the following problems?”; “Feeling down, depressed or hopeless”; “little interest or pleasure in doing things”; ‘feeling nervous or anxious or on edge,” and “not being able to stop or control worrying.” Respondents can respond “never”, “several days”, “more than half of the days” or “nearly every day”. Composite scores have been suggested to identify clinical distress: normal (0–2), mild (3–5), moderate (6–8) and severe (9–12), and cut-offs have been used. The PHQ-4 has been previously tested in humanitarian settings and among refugee populations: in the DRC (Visscher et al., 2018), Nigeria (Akingbade et al., 2023) and among Congolese (Pannetier et al., 2017) and Arab refugees (Jumaa et al., 2023). Internal consistency for the PHQ-4 at endline was adequate for women (Cronbach’s α = 0.77) and good for men (α = 0.82). Demographics assessed included education level, disability status, marital status, household composition, the identified index child’s gender and their disability level. Disability status was indicated on the Washington Disability Group Short Set Scale. In the context of this research, the term ‘index child’ refers to a designated child within the study population who serves as a focal point for data collection and analysis.

### Ethical considerations

The study procedures followed globally accepted ethical guidance on violence against women research (WHO, 2016). All participants provided informed consent at baseline and endline, and all interviews were conducted in private locations and with gender- and language-matched data collectors. All women received referral information to IRC case management services. The study was approved by the IRC Institutional Review Board (WPE #1.00.014) and the Comité National d’Ethique de la Santé-Direction Provincial du Sud-Kivu.

### Analysis

All data were analyzed separately for men and women in the stratified models. Descriptive frequencies and averages were generated from baseline demographics including participant age, education, disability, marital status and household composition, and index child gender and disability. Within each sex, baseline differences in demographics by treatment and control arms were also assessed using chi-square tests, and *t* tests to examine randomization success. Men’s data demonstrated a lack of balance for two demographics: (1) the number of people in household and (2) the child’s age.

Unadjusted and adjusted multilevel linear regression was implemented using xtmixed in Stata V15.0. Marginal regression models with errors clustered by participant group (n = 8) were used because they provided more conservative estimates. A time-X-treatment interaction term was constructed to assess the intervention impact as the independent variable on the dependent variable of endline mental health status. Adjusted models account for unbalanced baseline demographics of child age and number of household members. Although only men’s data exhibited a lack of balance of these demographics, both the women’s and men’s models were adjusted for women’s and men’s reporting of these variables, respectively, to improve precision in the stratified models. This secondary analysis had 80% power to detect a 1.05 mean change for women and 1.4 mean change for men in the PHQ-4 outcome. As this was an exploratory outcome, the study was powered to examine changes in violence; further details on sample size and power calculations are provided in Falb et al., 2023a.

## Results

### Women

A total of 198 women completed the baseline survey. There were no observable statistical differences across treatment arms for women based on the following demographics, suggesting that randomization was successful. Overall, the mean age (SD) of women in the study was 32.03 (8.71) (Table 1). 41.92% (n = 83) of women reported completing primary and 42.93% (n = 85) of women enrolled had not completed any education level. 21.21% (n = 42) of women enrolled reported no disability, while 60.10% (n = 119) reported having a mild disability and 18.96% (n = 37) of the enrolled women reported having a severe disability. Over half (52.02%; n = 103) of the enrolled women were currently married; 47.98% (n = 95) of the women reported “living as if married”, and 9.09% (n = 18) reported that they were currently displaced. The overall mean (SD) number of household members reported by the enrolled women was 7.21 (2.15), and the overall mean (SD) of their reported index child’s age was 10.01 (2.38). More enrolled women reported their index child’s gender as female (54.05%; n = 107) compared to male (45.96%; n = 91). Enrolled women reported an overall 27.78% (n = 55) of their index children having no disability, 47.98% (n = 95) as having some disability, and 24.24% (n = 48) as having moderate to severe disability.

**Table 1.** Descriptive demographics at baseline among women and men by treatment arm (N = 394)

	Women (N = 198)			Men (N = 196)		
Demographics	Overall	Safe at Home	Control	Overall	Safe at Home	Control
	Mean (SD) % (N)	Mean (SD) % (N)	Mean (SD) % (N)	Mean (SD) % (N)	Mean (SD) % (N)	Mean (SD) % (N)
Age (mean, SD)	32.03 (8.71)	32.41 (9.69)	31.62 (7.54)	35.88 (8.71)	36.12 (9.56)	35.62 (7.75)
Highest level of education						
Primary	41.92 (83)	36.98 (38)	47.37 (45)	44.39 (87)	44.55 (45)	44.21 (42)
Secondary and above	15.16 (30)	17.47 (18)	12.63 (12)	43.37 (85)	43.56 (44)	43.16 (41)
None	42.93 (85)	45.63 (47)	40.00 (38)	12.24 (24)	11.88 (12)	12.63 (12)
Disability level						
No disability	21.21 (42)	22.33 (23)	20.00 (19)	39.29 (77)	36.63 (37)	42.11 (40)
Mild disability	60.10 (119)	60.19 (62)	60.00 (57)	42.35 (83)	43.56 (44)	41.05 (39)
Moderate/severe disability	18.69 (37)	17.48 (18)	20.00 (19)	18.37 (36)	19.80 (20)	16.84 (16)
Marital status						
Currently married	52.02 (103)	50.49 (52)	53.68 (51)	67.35 (132)	65.35 (66)	69.47 (66)
Living as if married	47.98 (95)	49.51 (51)	46.32 (44)	32.65 (64)	34.65 (35)	30.53 (29)
Currently displaced	9.09 (18)	12.62 (13)	5.26 (5)	4.08 (8)	4.95 (5)	3.16 (3)
Household composition						
Number of household members (mean, SD)	7.21 (2.15)	6.98 (2.11)	7.45 (2.17)	7.20 (2.25)	**6.79 (1.98)**	7.64 (2.43)**
Index child age	10.01 (2.38)	9.89 (2.32)	10.13 (2.45)	9.82 (2.31)	**9.50 (2.35)**	10.16 (2.23)*
Index child gender (%)						
Boy	45.96 (91)	43.69 (45)	48.42 (46)	44.39 (87)	47.82 (48)	41.05 (39)
Girl	54.04 (107)	56.31 (58)	51.58 (49)	55.61 (109)	52.48 (53)	58.95 (56)
Index child disability level						
No disability	27.78 (55)	30.10 (31)	25.26 (24)	45.41 (89)	45.54 (46)	45.26 (43)
Some disability	47.98 (95)	48.54 (50)	47.37 (45)	44.90 (88)	42.57 (43)	47.37 (45)
Moderate/severe disability	24.24 (48)	21.36 (22)	27.37 (26)	9.69 (19)	11.88 (12)	7.37 (7)

** p < .01.

* p < .05.

### Men

There was a total of 196 men randomized to either the Safe at Home intervention or the control group. Men in the study sample were also balanced at baseline, with the exception of the index child age and the number of household members. Overall, the mean age (SD) of men in the study was 35.88 (8.71) (Table 1). 44.39% (n = 87) of the enrolled men overall reported completing primary education, 38.78% (n = 76) reported completing secondary education, 4.08% (n = 8) reported completing university and 0.51% (n = 1) reported completing vocational education. Overall, 12.24% (n = 24) of the enrolled men reported having no education level completed. 39.39% (n = 77) of the enrolled men overall reported no disability, while 42.35% (n = 83) reported a mild disability, and 18.37% (n = 36) reported a moderate or severe disability. Over half of the men enrolled reported themselves as being currently married (65.35%; n = 66). In addition, 32.65% (n = 64) of the enrolled men reported themselves as “living as if married”, and an overall 4.08% (n = 8) reported themselves as currently displaced. The overall mean (SD) number of household members reported by the enrolled men were 7.20 (2.25), and the overall mean (SD) of their reported index child’s age was 9.82 (2.31). More enrolled men reported their index child’s gender as female (55.61%; n = 109) compared to male (44.39%; n = 87). Overall, 45.54% (n = 89) of the enrolled men reported their index child as having no disability, 44.90% (n = 88) reported their index child as having some disability, and 9.69% (n = 19) reported their index child as having a moderate to severe disability.

### Mental health among women at endline

One hundred ninety-nine women completed the endline. The Safe at Home group (N = 102) had a mean mental health score of 3.55 (SD 3.04) (Table 2). The control group (N = 97) had a mean of 4.56 (SD 2.64). The intervention effect estimates showed a statistically significant difference in mental health for Safe at Home in both the basic model (β = −1.01, 95% CI [−1.85, −0.17], p = 0.018) and the adjusted model (β = −0.96, 95% CI [−1.85, −0.07], p = 0.035).

**Table 2.** Estimated effect of the intervention on PHQ-4 score among men and women, controlling for clustering by group. Adjusted model controls for index child age and number of household members

	Summary statistics at endline		Intervention effect estimate*	
Group	Safe at Home	Control	Basic model	Adjusted model
	Mean (SD)	Mean (SD)	β (95%CI), p-value	β (95%CI), p-value
Women^1^ (n = 199)	3.55 (3.04) N = 102	4.56 (2.64) N = 97	**−1.01 (−1.85, −0.17), p = 0.018**	**−0.96 (−1.85, −0.07), p = 0.035**
Men^2^ (n = 192)	5.11 (3.73) N = 96	5.23 (3.45) N = 96	−0.12 (−1.32, 1.06) P = 0.840	−0.13 (−1.43, 1.17) P = 0.849

*Notes: 1. ICC for women = 9.38e^−12^. 2. ICC for men = 0.018.*

### Mental health among men at endline

At endline, 192 men completed the survey. The Safe at Home group (N = 96) had a mean mental health score of 5.11 (3.73) (Table 2). The control group (N = 96) had a mean of 5.23 (3.45). The intervention effect estimates did *not* show a statistically significant difference in mental health for both Safe at Home intervention and control. In the basic model, the β coefficient for mental health in the Safe at Home group was −0.12 (95% CI [−1.32, 1.08], p = 0.840), and in the adjusted model, the β coefficient was −0.13 (95% CI [−1.43, 1.17], p = 0.849).

## Discussion

The present analysis documents that an effort to prevent family violence and improve family well-being in a conflict-affected setting in eastern DRC may have improved mental health for women, but not for men. As only endline measures were available, this interpretation assumes the balance between the intervention and control arms within sexes at baseline. This may be assumed as there were no observable differences in outcomes across treatment groups in the full trial and limited statistical differences for demographics. Overall, these differential findings between women and men may be due to family violence prevention resulting in less victimization for women; therefore, the expectation of improvement for men is not necessarily expected within the family violence prevention model. However, the differential findings may also be due to how the Safe at Home family violence prevention intervention could have influenced mental health through different pathways, such as improved social support of group participants, which may have been particularly important for female participants. Female participants may have expanded and strengthened social networks and bolstered a sense of solidarity. Additionally, given Safe at Home’s direct impact on reductions of IPV, overall well-being of women may have improved, alongside other dimensions of caregiver and partner dynamics (Falb, et al., 2023b). Future research on the relationship between violence prevention and mental health for women should assess pathways and dyadic relationships in a fully powered study. Importantly, when adjusted for demographic differences, the impact of the Safe at Home intervention on power sharing behaviors among women was statistically significant. This finding supports the growing feminist mental health literature that advocates for the inclusion of women in power-sharing systems as a method to promote gender inclusivity and mental health (Byrne, 2020). Future longitudinal research should investigate the association between mental health and power sharing behaviors, social support and networks and other pathways for women in a fully powered study that includes these more comprehensive measures. Additionally, feminist-grounded violence prevention interventions may benefit from a more intentional combined approach to magnify mental health components and pathways for women. Such programming may benefit from lessons learned from interventions that originate in mental health and psychosocial programming that have sought to integrate violence prevention elements (Tol et al., 2017; Greene et al., 2021, 2022).

The lack of statistical change for improvements in men’s mental health may be attributed to programmatic or research design reasons. First, changes seen for women could have been due to a dosage effect. While men and women participated in an average of 13 sessions, the men’s curriculum was 6 sessions longer than the women’s curriculum. Lower intervention adherence relative to women may have affected possible mental health benefits, as exposure to a greater percentage of the intended content may improve intervention effects overall. Furthermore, it should be noted that the COVID-19 pandemic may have further decreased participation among men because some men lost their livelihoods and had to travel frequently for daily work (Falb et al., 2023b). Women may have participated more consistently in the intervention because they have fewer public spaces to build community, and the Safe at Home intervention may have provided women with the benefit of social support. Second, the hypothesized pathways by which the intervention could impact women’s and men’s mental health differed. As stated previously, women’s mental health may have benefited from elements to mitigate violence, including improving relationship quality, psychosocial support and positive parenting strategies as well as violence reduction itself. It may be that men’s mental health is not as significantly impacted by these same elements. Interventions may need to go beyond light-touch trauma-informed stress management techniques provided in Safe at Home to improve men’s mental health.

Third, such findings may also be attributed to the complexities of the male experience within humanitarian settings (Courtenay, 2000, Hart, 2008, Jaji, 2009, Kabachnik et al., 2013). For example, there have been suggested vulnerabilities unique to the “provider and protector” role that men traditionally play, suggesting that this role is threatened by the uncertainty and lack of self-determination conflict-afflicted settings provide (Affleck et al., 2018). As previously demonstrated through the literature, these feelings are often externalized in avoidant or violent behavior characteristic of post-traumatic stress symptoms (Johnson et al., 2010). As such, the family violence prevention model may not have addressed such gendered determinants of mental health, and future research should aim to address these complexities. For example, it is plausible that incorporation of an economic well-being component to Safe at Home would have magnified effect on both caregivers’ mental health outcomes (Falb et al., 2014; Hammad and Tribe, 2020), as previous work in DRC has also documented how economic programming may improve adolescent and adult mental health (Glass et al., 2017, 2020) as well as moderate the relationship between conflict-related traumatic events and mental health (Glass et al., 2014).

Importantly, practitioner insights on men’s mental health changes in the program differ from our quantitative findings. For example, implementing practitioners indicated that men’s distress reduces when men’s wives and children are in a good mental state, an outcome that is consistent with the findings of this study for women. These insights from practitioners may not have been accurately captured in our quantitative findings due to sample size issues, previously mentioned dosage effects, and the selection of measures for exploratory analysis (i.e., PHQ-4). Future studies should explore these qualitative insights from practitioners as a framework from which mental health changes can be explicitly ascertained as an outcome of family violence prevention.

The findings of this analysis should be interpreted with limitations in mind. First, the Safe at Home intervention was not designed to address mental health, and therefore there was no mental health measure included at baseline. Nonetheless, given the overall balance of treatment arms and the ability to adjust for two unbalanced demographic variables, we can plausibly assume that the PHQ-4 was also balanced at baseline. However, this lack of baseline assessment impedes the study team’s ability to adjust for baseline mental health status, which would have helped to further explain the differences in mental health outcomes between men and women. For example, it may be plausible that men had better mental health than women at baseline and may be more subject to a ceiling effect that limits our ability to statistically examine these changes, although this interpretation is unlikely given men indicated worse mental health than women at endline overall. It is important to note that within-gender mental health outcomes may not have been impacted by the lack of baseline data; however, the lack of baseline data presents limitations in the ability to more thoroughly compare results between gender groups. Additionally, the PHQ-4 is a lean, generalized measure of mental health (Kroenke et al., 2009). A more detailed exploration of mental health outcomes, including contextually relevant conceptualizations of post-traumatic stress, anxiety and depression (Betancourt et al., 2011; Higson-Smith and Eagle, 2017; Parcesepe et al., 2023) could provide a more comprehensive understanding of the mechanisms underlying the observed effects. As such, due to the lack of validation of the PHQ-4 cutoffs in the DRC context, we are unable to extrapolate the clinical significance of these findings. Finally, given that the present analysis does not include baseline data or fully captures all potential pathways between Safe at Home and mental health outcomes, we recommend potential mediator analyses in future research. Although caregivers also reported decreases in the use of harsh discipline and improvement in parenting skills that could benefit children, the study was also not able to collect mental health data from children for ethical and logistical reasons. Future research should also explore the potential intergenerational impact of family violence prevention programs on caregiver mental health and subsequent improvement in children’s mental health (Howarth et al., 2016; Gartland et al., 2019; Lünnemann et al., 2019; Murray et al., 2020). This may extend previous work in eastern DRC that documented the importance of parental mental health in magnifying effects on the mental health of children and adolescents (Glass et al., 2018). Nonetheless, this study provides essential data to inform future trials and provides a rationale for increased sample size and more robust measurement of mental health outcomes.

## Conclusions

Ultimately, this exploratory analysis provides evidence of the potential for a family violence prevention model to improve women’s mental health in a low-resource, conflict-affected setting, although further research is needed to understand the impact on men’s mental health. Family violence prevention approaches may be added to the growing evidence of both more clinical interventions derived from cognitive behavioral therapy interventions (Bass et al., 2013; Glass et al., 2014), and economic-based programming, such as village savings and loans or poverty alleviation schemes, to improve mental health among conflict-affected populations (Green et al., 2016; Annan et al., 2017; Zaneva et al., 2022). Further research is needed to understand the dynamic and cyclical relationship between interrupting experiences of violence and poor mental health to promote resilience and well-being for families in humanitarian settings across generations.

## Data Availability

Data are available on request to the principal investigator.
