# Supplementary material for: Effectiveness of a family violence prevention program on mental health outcomes for adult men and women in North Kivu, Democratic Republic of Congo: Insights from a pilot trial
Source: Glob Ment Health (Camb). 2024 Oct 24;11:e99. doi: 10.1017/gmh.2024.98 (PMC11504927; doi:10.1017/gmh.2024.98)
Supplement: Peer review history

# Editor report: Recommendation: Effectiveness of a family violence prevention program on mental health outcomes for adult men and women in North Kivu, Democratic Republic of Congo: Insights from a pilot trial — R0/PR2

**DOI:** 10.1017/gmh.2024.98.pr2

**Affiliations:** School of Public Health, Universidad del Valle, Colombia

Dear authors: 

Please, follow the reviewers' suggestions completing the corrections. 

As mentioned by reviewers, it’s important to highlight and discuss the strong methodological limitations of the PHQ-4 instrument. 

Best regards.

# Editor report: Decision: Effectiveness of a family violence prevention program on mental health outcomes for adult men and women in North Kivu, Democratic Republic of Congo: Insights from a pilot trial — R0/PR3

**DOI:** 10.1017/gmh.2024.98.pr3

**Affiliations:** Johns Hopkins University Bloomberg School of Public Health, United States

No accompanying comment.

# Editor report: Recommendation: Effectiveness of a family violence prevention program on mental health outcomes for adult men and women in North Kivu, Democratic Republic of Congo: Insights from a pilot trial — R1/PR5

**DOI:** 10.1017/gmh.2024.98.pr5

**Affiliations:** School of Public Health, Universidad del Valle, Colombia

Dear authors: 

Your manuscript requires a Minor Revision, especially on to the important methodological issues addressed by the reviewers. 

Best regards.

# Editor report: Decision: Effectiveness of a family violence prevention program on mental health outcomes for adult men and women in North Kivu, Democratic Republic of Congo: Insights from a pilot trial — R1/PR6

**DOI:** 10.1017/gmh.2024.98.pr6

**Affiliations:** Johns Hopkins University Bloomberg School of Public Health, United States

No accompanying comment.

# Editor report: Recommendation: Effectiveness of a family violence prevention program on mental health outcomes for adult men and women in North Kivu, Democratic Republic of Congo: Insights from a pilot trial — R2/PR8

**DOI:** 10.1017/gmh.2024.98.pr8

**Affiliations:** School of Public Health, Universidad del Valle, Colombia

Dear authors: The reviewers indicates that you have clearly addressed the remaining issues, thus, the manuscript is accepted. Best regards.

# Editor report: Decision: Effectiveness of a family violence prevention program on mental health outcomes for adult men and women in North Kivu, Democratic Republic of Congo: Insights from a pilot trial — R2/PR9

**DOI:** 10.1017/gmh.2024.98.pr9

**Affiliations:** Johns Hopkins University Bloomberg School of Public Health, United States

No accompanying comment.
